# Pull-Out Progressive Damage and Failure Analysis of Laminated Composite Bolted Joints

**DOI:** 10.3390/ma17235747

**Published:** 2024-11-24

**Authors:** Zhaowei Zeng, Qixiang Fan, Feng Liao, Gang Liu, Jianwei Yan

**Affiliations:** 1 Department of Engineering Graphics and Software, East China Jiaotong University, Nanchang 330013, China; zhwzeng@ecjtu.edu.cn (Z.Z.); liug@ecjtu.edu.cn (G.L.); 2 School of Civil Engineering and Architecture, East China Jiaotong University, Nanchang 330013, China; 2023018085901074@ecjtu.edu.cn (Q.F.); tyanjianwei@jnu.edu.cn (J.Y.); 3 Low Speed Aerodynamics Institute, China Aerodynamics Research and Development Center, Mianyang 621051, China; 4 Jiangxi Key Laboratory of Disaster Prevention-Mitigation and Emergency Management, East China Jiaotong University, Nanchang 330013, China

**Keywords:** composite bolted joints, progressive damage, failure analysis

## Abstract

Laminated composite bolted joints are increasingly used in the aerospace field, and their damage and failure behavior has been studied in depth. In view of the complexity and stability requirements of laminated composite bolted structures, accurate prediction of damage evolution and failure behavior is significant to ensure the safety and reliability of the structures. In this paper, a novel asymptotic damage model is developed to predict the damage process and failure behavior of laminated composite bolted joints. In this model, the modified Puck criterion and the maximum shear stress criterion are used for fiber yarns. The parabolic yield criterion is adopted for the matrix, and the fiber fracture, inter-fiber fracture and matrix fracture are considered at the microscopic level. The pull-out strength and progressive failure behavior of countersunk and convex bolted joints structures are predicted by using the proposed model, and the corresponding experimental studies are carried out. The results show that the prediction results are in good agreement with the experimental data, which verifies the reliability of the model. Additionally, the effects of different structural parameters (thickness and aperture) on the progressive damage and failure behavior during pull-out is analyzed by the proposed model, and correction factors of pull-out strength are obtained, which provides a powerful tool for the design, analysis and progression of laminated composite bolted joint structures.

## 1. Introduction

The wide application of advanced fiber reinforced composite structures reflects its superior performance in many industrial fields [1]. The high specific stiffness and strength make these materials lighter and thinner than traditional materials when carrying the same load, which effectively reduces the overall structural weight and improves the carrying efficiency. In the field of aerospace, the joints structure of composite materials has been a hot topic studied by many researchers. The bolted joint is one of the most common and vital connection methods for the assembly of structural components, which can effectively connect the composite structure of different materials, provide excellent mechanical properties and reliability, and ensure the safe and stable operation of aerospace spacecraft [2].

In recent decades, research on laminated composite bolted joints was frequently reported. Vadean et al. [3] developed a fast and accurate numerical calculation method for the bolted joints of very large bearings, which improves the calculation efficiency and accuracy. Chang et al. [4] proposed a progressive damage model for bolted joints of laminate composites, which can effectively simulate the progressive damage and failure behavior of bolted joints under tensile or shear loads. Through experimental research and numerical simulation, Tang et al. [5] established a nonlinear contact model of braided composite bolted joints based on Hashin failure criteria, which can effectively predict the failure mode and load-displacement curve of bolted joints. Ireman et al. [6] constructed a 3D finite element model of bolted composite joints, which can be used to determine the non-uniform stress distribution of composite laminate in the thickness direction near the bolt hole. Gray et al. [7] studied the influence of thickness and laminate taper on the stiffness, strength and secondary bending of single-lap and single-bolt countersunk composite joints. Taheri-Behrooz et al. [8] developed an analytical method for considering the effect of component material nonlinearity on the load distribution of single-stud multi-bolt composite joints and offered a computational tool for considering material nonlinearity. In addition, it was observed that an increase in the degree of nonlinearity of the member material results in an increase in the load transferred by the outer bolt of the joint and a decrease in the load transferred by the inner bolt. Xiao et al. [9,10] constructed a progressive model to predict the experimental results based on the mixed failure criteria of Hashin and Yamada Sun and the degradation model. Ni et al. [11] presented a custom element development method based on the ABAQUS software platform. The 2D finite element model created by this method can simulate the complete nonlinear mechanical characteristics of the bolted joints of composite materials. The results of this method are reliable and the calculation efficiency is high. Egan et al. [12] established a progressive damage and failure model of countersunk composite joints under a quasi-static load by using the 3D explicit finite element analysis method. Liang et al. [13] proposed a single bolt equivalent model. The parameters in the Johnson-Cook model can be calibrated effectively by using the equivalent model. Using the improved J-C model, the bolted joints under tensile, flexural and shear combined loads were further studied. Pearce et al. [14] adopted a laminated modeling method to simulate the interaction between layering and lamination failure under complex dynamic loads, providing a simulation tool for the pull-through failure of composite bolted structures. Kelly et al. [15] conducted experiments and numerical analyses to investigate the performance of composite laminates under transverse loading, uncovering two significantly different macroscopic mechanical failure modes. Stocchi et al. [16] carried out a comprehensive finite element study on composite bolt joints with countersunk fasteners. Veisi et al. [17] conducted an in-depth numerical simulation and experimental study on the strength of composite double-bolt joints and the progressive damage behavior of bearings. Sharos [18] developed a finite element simulation of composite bolt joints, which had been verified by experiments with high efficiency and fidelity and can make real-time decisions. It was found that the dynamic load distribution is temporal and rate-dependent, and the stress wave caused the load unbalance to increase by 85%, which affected the joint design. Giannopoulos et al. [19] considered the influence of preload torque on the bolt joints performance of composite materials. The static strength increased with the increase of preload torque, and the preload level has an upper limit. The failure position of joints with high torque was behind the gasket, and the preload level and diameter of the gasket affect the damage modes. Long displacement before the failure of specimens with low preload torque was beneficial to the design. Montagne et al. [20] and Qu et al. [21] established progressive damage models considering the Hashin criteria and material exponential degradation. Combined with experiments, they studied single-lap bolt joints with different end distances and revealed the effects of forced assembly on the stress distribution and load damage of single-lap countersunk and non-countersunk composite bolt joints. Shan et al. [22] proposed a two-material property method to describe the mechanical properties of unidirectional composite layers. Combined with the Hashin failure criterion and material degradation model based on micromechanics, the progressive damage model of a typical double-lash bolted connection was successfully established. Shan et al. [23] proposed a numerical framework based on characteristic curves, established a three-dimensional finite element model considering the effect of moisture and heat, and analyzed the bolt load distribution in a multi-bolt connection. The failure form and strength of the composite joint were obtained by using a new characteristic curve considering the influence of moisture and heat. Guo et al. [24] and Shi et al. [25] carried out numerical calculation and experimental analysis on the connection strength and failure mode of bolted composite laminates based on the material failure criteria. It was found that different bolt arrangements have significant influence on the failure strength of bolted laminates. Lin et al. [26], based on the high-fidelity finite element model with detailed threaded structure to obtain accurate initial assembly states, proposed a method that can accurately analyze the progressive failure and static strength of composite bolted bearings under different initial assembly parameters, and analyzed the formation and evolution mechanism of the tension yield phenomenon during assembly, and the influence of two key assembly parameters, washer type and bolt initial tension, on the bearing strength of composite bolt connection. Zhao et al. [27] proposed a concatenated generation model, bolt tightening generation adversarial network, to predict the stress field of bolt connections of composite materials during assembly. This network improved the accuracy of stress field prediction by extracting the understanding of multi-scale features through skip connections and the attention mechanism. After training, the model can complete a prediction in only 6.1 s, and the prediction efficiency was significantly improved compared with the finite element analysis. The efficient prediction of this model was conducive to the digital twin modeling of the assembly line and the effective control of assembly quality, and provides a powerful tool for assembly design and analysis.

To sum up, the existing research mainly focuses on the static strength analysis and macro damage of bolted structures, while the dynamic strength analysis and micro damage mechanism of composite materials received less attention; the pull-out performance of composite bolted joints, especially, is rarely reported. Composite bolted joints have poor normal bearing capacity and are prone to pull-out failure under out-of-plane loads, so it is necessary to analyze the pull-out progressive damage and failure of composite structures.

Typically, the failure modes of laminated composite bolted joints usually cover the aspects of net tension, shear, bearing and so on. The net tension and shear failure happen suddenly and result in catastrophic destruction, which belongs to brittle failure modes. In comparison, bearing failure is a slow and gradual mode of failure, and is commonly referred to as plastic failure. Therefore, when designing laminated composite bolted joints, it is crucial to consider the structural parameters of the bolts and material plates in order to achieve the desired ultimate failure mode of bearing failure. By optimizing these parameters, one can ensure that the joint fails in a gradual and predictable manner, avoiding catastrophic failures occurring with other types of failure modes. Laminated composite bolted joints that fail in the bearing failure mode can have their failures predicted through early detection, allowing for the setting of a certain safety factor for the working strength of bolted joints. This greatly reduces the risk of safety accidents. Load-bearing failure is a common failure mode in practical engineering, especially in the process of fastener pull-out failure. Therefore, the progressive damage and failure process of materials and structures should be fully considered when the finite element model is established.

This paper develops a three-dimensional detailed finite element progressive damage model to predict the damage in composite bolted joints. Tensile pull-off tests were performed on protruding-head bolt and countersunk bolt joints, and the experimental data are compared with the predicted results from the model. The good agreement between the results validates the reliability of the model. The model is divided into two parts: an initial damage model and a progressive degradation model. The initial damage accumulation model is constructed by combining the maximum stress criterion with the Puck criterion. A progressive degradation model is then employed to describe the process from initial damage to ultimate failure. Furthermore, a continuous degradation factor is utilized to characterize the failure behavior of the laminated plates. Once the occurrence of initial damage is detected, the performance of the damaged elements degrades, leading to a gradual reduction in the load-bearing capacity of the bolted joint structure. The three-dimensional progressive damage finite element model established in this paper has demonstrated significant effectiveness in predicting the damage and failure behavior of laminated composite bolted joints. Compared with the Hashin, LaRC02 and other criteria, the Puck criteria put forward the concept of action plane proposal on the basis of them; they represent the influence of transverse compression on the shear strength of matrix, and fully consider the shear damage of component materials. The influence of shear failure must be fully considered in the progressive damage and failure process of bolted joint structures. By incorporating the Puck criterion, the model is able to comprehensively consider the effects of fracture planes in judging material damage, thereby more accurately simulating the actual failure process of composite materials. This innovative application not only enhances the prediction accuracy of the model but also provides a new perspective and tool for studying the mechanical properties of composite laminated bolted joints. But this method needs extensive experimental results to support in the early stage.

## 2. The Model for Initial Damage

### 2.1. Damage Initiation

Composites often have initial damage such as micromechanical defects or cracks. When subjected to external loads, the composite material will not immediately lose its bearing capacity; however, as the load increases, the initial damage can become catastrophic. In view of this, it is necessary to construct reasonable and accurate initial damage criteria to predict the location of compound damage. Since Puck theory can predict the location and direction of damage, Puck theory is selected as the initial damage criterion of single-layer plate, and the damage conditions of single-layer plate in different directions are analyzed. In the tensile damage model, the modified Puck criterion [28,29] and the maximum shear stress criterion are used as the damage initiation criterion, the parabolic yield criterion [30] and the maximum shear stress criterion. The failure criteria of fiber yarns are

(1) $$\left\{\begin{array}{c} f_{E,1}^{t}=\frac{1}{x_{p}^{c}}[E_{P}\varepsilon_{1}+1.1v_{PV}(\sigma_{2}+\sigma_{3})]\sigma_{1}\geq 0\\ f_{E,1}^{c}=\frac{-1}{x_{p}^{c}}[E_{P}\varepsilon_{1}+1.1v_{PV}(\sigma_{2}+\sigma_{3})]\sigma_{1}<0 \end{array}\right.,$$

(2) $$f_{E,2}^{t,c}=\sin^{2}\alpha_{fp}+\cos^{2}\alpha_{fp}f_{E}^{t,c}\left(\alpha_{fp}\right),\left\{\begin{array}{c} t:\sigma_{n}(\alpha )\geq 0\\ c:\sigma_{n}(\alpha )<0 \end{array}\right.,$$

(3) $$f_{E,3}^{t,c}=\cos^{2}\alpha_{fp}+\sin^{2}\alpha_{fp}f_{E}^{t,c}\left(\alpha_{fp}\right),\left\{\begin{array}{c} t:\sigma_{n}(\alpha )\geq 0\\ c:\sigma_{n}(\alpha )<0 \end{array}\right.,$$

(4) $$f_{E,h}=\left|\frac{\tau_{k}}{X_{k}}\right|,k=\left\{VP,VV\right\},\hbar =\left\{4,5,6\right\},$$

where Equation (1) shows the stress exposure of fiber breakage, and (4) is mainly caused by stress parallel to the fiber. Formulas (2) and (3) describe the stress exposure of inter-fiber fractures. Fractures occur only when the stress exceeds $f_{E}\geq$ 1. The superscripts t and c represent tension and compression forces, respectively. The subscripts P, V, VP and VV indicate vertical, horizontal, in-plane and out-of-plane directions, respectively. E, X and $\alpha_{fp}$ are the modulus, strength and breaking angle of the fiber yarn, respectively. *υ_PV_* represents the main Poisson quantification of fiber yarns. The failure criteria of the matrix are as follows:

(5) $$m_{E}^{tc}=\pm \frac{2{\left(\sigma_{m,1}-\sigma_{m,2}\right)}^{2}+{\left(\sigma_{m,2}-\sigma_{m,3}\right)}^{2}{\left(\sigma_{m,3}-\sigma_{m,1}\right)}^{2}+\left(\sigma_{m,1}+\sigma_{m,2}+\sigma_{m,3}\right)\left(X_{m}^{c}-X_{m}^{t}\right)}{X_{m}^{c}X_{m}^{t}},$$

(6) $$m_{E,s}=\left|\frac{\tau_{s}}{X_{s}}\right|,$$

Here, $I_{1}=\sigma_{m,1}+\sigma_{m,2}+\sigma_{m,3}\geq 0$ indicates tension, otherwise compression. Subscript m represents matrix and $X_{s}$ indicates the shear strength of matrix. Fracture only occurs when the stress exposure of matrix fracture $m_{E}$ ≥ 1.

### 2.2. Damage Evolution

As aforementioned, the fiber yarns and matrix begin to damage when the stress exposures $f_{E}$ and $m_{E}$ are equal to zero. With the accumulation of damage, the amount of stress exposure increases gradually. Assuming that T is the period from the beginning of damage to the complete failure of the component material, the relationship between stress exposure and the damage threshold factors of the fiber yarn and the matrix can be expressed as follows:

(7) $$r_{j}^{t,c}=\max\left\{1,\underset{s\in \left[0,T\right]}{max}\;\left\{f_{E,j}^{t,c}\right\}\right\},j=\left\{1,2,3\right\},$$

(8) $$r_{h}=\max\left\{1,f_{E,h}\right\},\hbar =\left\{4,5,6\right\},$$

(9) $$r_{m}^{t,c}=max\left\{1,\underset{s\in \left[0,T\right]}{max}\;\left\{m_{E,j}^{t,c}\right\}\right\},$$

(10) $$r_{s}=\max\left\{1,m_{E,s}\right\},$$

The exponential damage evolution law is adopted for fiber yarns and matrix. The form of the damage variable is as follows:

(11) $$d_{j}^{t,c}=1-\frac{1}{r_{j}^{t,c}}\exp\left[A_{j}^{t,c}\left(1-r_{j}^{t,c}\right)\right],j=\left\{1,2,3\right\},$$

(12) $$d_{h}=1-\frac{1}{r_{h}}\exp\left[A_{h}\left(1-r_{h}\right)\right],\hbar =\left\{4,5,6\right\},$$

(13) $$d_{m}^{t,f}=1-\frac{1}{r_{m}^{t,c}}\exp\left[A_{m}^{t,c}\left(1-r_{m}^{t,c}\right)\right],$$

(14) $$d_{s}=1-\frac{1}{r_{s}}\exp\left[A_{s}\left(1-r_{s}\right)\right],$$

Here, $A_{j}^{t,c}$, $A_{h}$, $A_{m}^{t,c}$ and $A_{s}$ are the parameters of damage evolution of the fiber yarns and matrix. These parameters can be obtained according to the following formula on Bazant’s crack zone theory.

(15) $$A_{Z}=\frac{2bX_{2}^{2}}{2E_{Z}G_{Z}-2bX_{2}^{2}},Z=\left\{1,2,3,4,5,6,m,s\right\},$$

where $G_{Z}$ represents the energy release rate, and the values of $A_{Z}$ are related to the characteristic length of the element b. Therefore, the compliance matrixes with damage factors of fiber yarns and matrix go to

(16) $$S_{f}\left(d_{f}\right)=\begin{array}{cccccc} \frac{1}{\left(1-d_{1}\right)E_{p}} & \frac{\upsilon_{VP}}{E_{p}} & \frac{\upsilon_{VP}}{E_{p}} & & & \\ & \frac{1}{\left(1-d_{2}\right)E_{V}} & \frac{\upsilon_{VV}}{E_{V}} & & 0 & \\ & & \frac{1}{\left(1-d_{3}\right)E_{V}} & & & \\ & & & \frac{1}{\left(1-d_{4}\right)G_{VP}} & & \\ & sym. & & & \frac{1}{\left(1-d_{5}\right)G_{VV}} & \\ & & & & & \frac{1}{\left(1-d_{6}\right)G_{VP}} \end{array},$$

where *E_p_*, *E_V_*, *G_VP_*, *G_VV_*, *ν_VP_* and *ν_VV_* are elastic constants of fiber and *d_i_* (*i =* 1~6) are damage factors of different damage forms. Damage factor *d*_1_ is relative to the longitudinal rupture of fiber. *d*_2_ and *d*_3_ are relative to the transverse rupture of the single-layer plate. *d*_4_ and *d*_6_ are relative to fiber rupture and inter-fiber rupture of the single-layer plate. *d*_5_ is influenced by the inter-fiber rupture of the single-layer plate [31].

## 3. The Termination Condition of Damage Evolution

**Figure 1:**
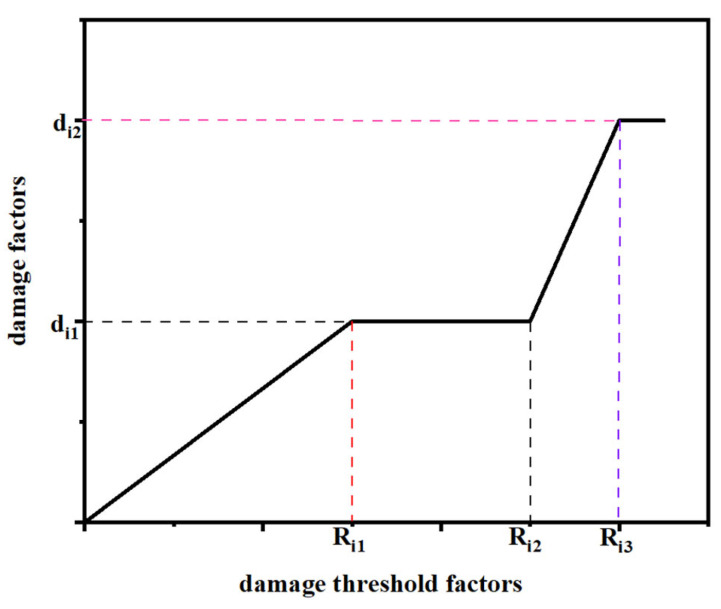
Damage factors evolution process.

As shown in Figure 1, the damage process is controlled by the damage factor *d_i_* and the damage threshold factor *R_i_*. When the material is subjected to a certain compressive load, the performance of the material begins to deteriorate. The performance degradation of the material can be divided into two stages. In the first stage, the material performance will gradually degenerate to a critical value, and eventually maintain a constant value. More details can be found in reference [31].

Stage 1:

(17) $$d_{i}\leq d_{i,first},$$

In Stage 2, the stiffness of the material reaches a maximum, reaches $d_{i,first}$, and stays at this value. As the load increases, the stiffness of the material will continue to deteriorate, which can be expressed as follows:

Stage 2:

(18) $$d_{i}=d_{i,second}\ldots \;when\;R_{ic}>R_{icx}\;or\;R_{it}>R_{itx},$$

where $R_{icx}$ and $R_{itx}$ are the second critical conditions. $d_{i,second}$ > $d_{i,first}$ denotes further degradation of material properties. This degradation is not linear but is affected by a combination of factors. For example, the change of microstructure in material, temperature and humidity in external environment may change the rate and degree of stiffness degradation. The key parameters of damage evolution are shown in Table 1.

At the same time, the strength of the material may change accordingly in this process. If the degradation rate of material stiffness is too quick, it may cause the failure of the material before the expected service life. Therefore, it is of great significance to study the variation of stiffness in the second stage for accurately evaluating the reliability and durability of materials.

## 4. Modeling and Finite Element Implementation

**Figure 2:**
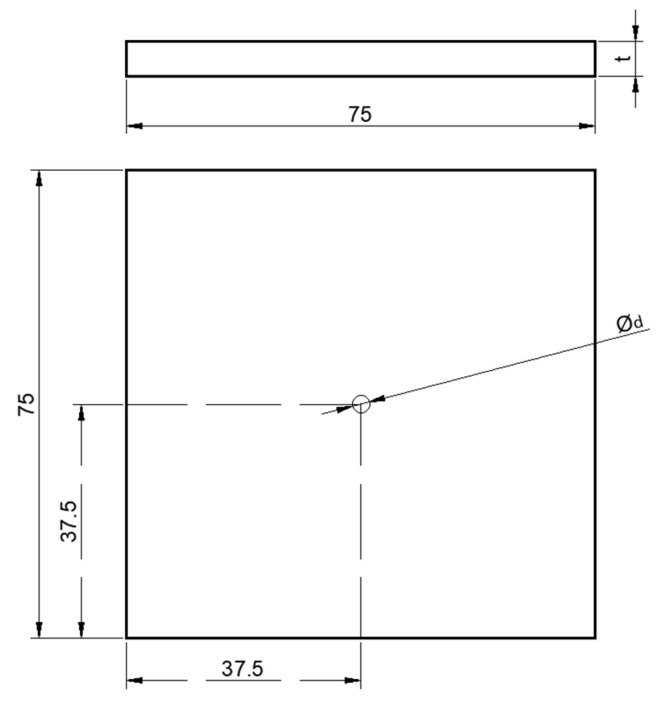
Schematic diagram of laminate.

**Figure 3:**
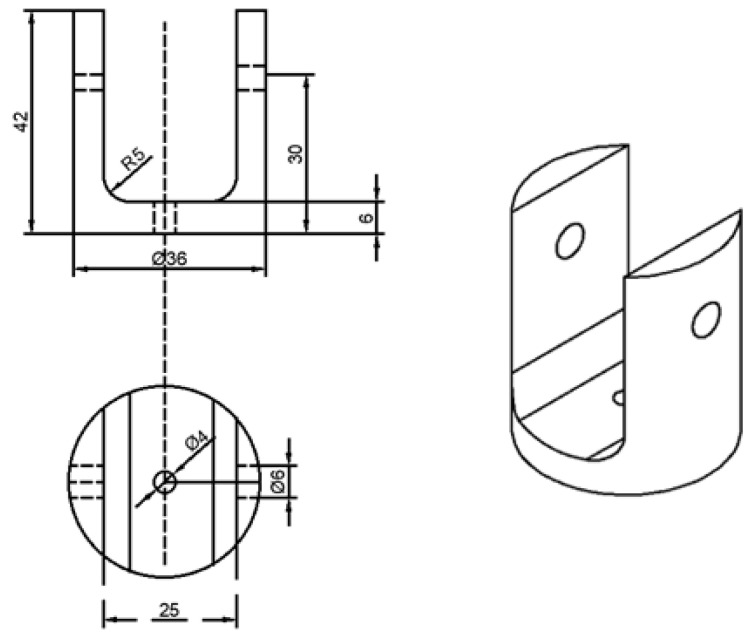
Diagram of constraint dimensions.

**Figure 4:**
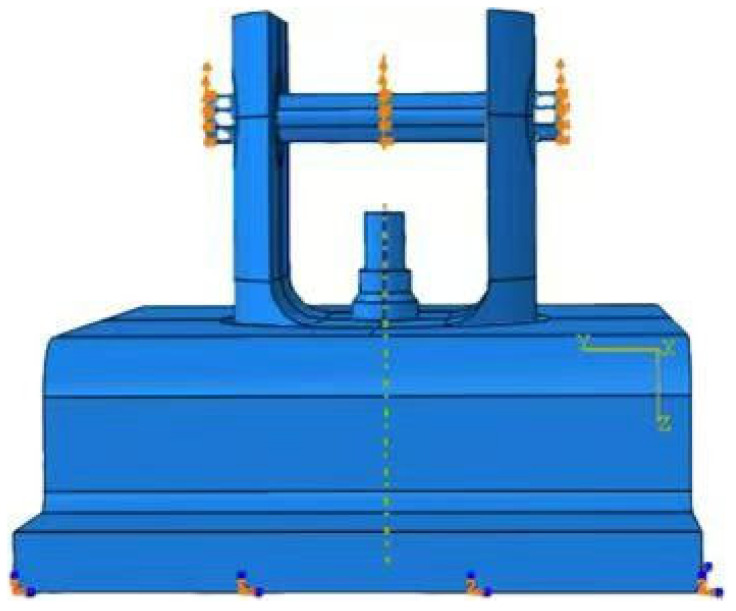
Load conditions.

The geometric dimensions of the material plate refer to the standard of ASTM D7332-2009B [32], as shown in Figure 2. In the part of fastener pull-out analysis, two basic simulation models of single-hole single shear countersunk head, pull-out countersunk head and pull-out raised head are needed. Using the same damage criterion and damage evolution model as the analysis of extrusion strength, all analysis ends with a 30% reduction of load. The restraint device for the test is shown in Figure 3, and the test loading mode is shown in Figure 4.

When the bolted joints are subjected to axial tensile load, there will be an interactive force between the bolt and the connected parts. When the load gradually increases to a certain extent, possible failure modes such as yielding, fracture or detachment may occur in the bolted joints. The mechanical properties of bolted joints can be analyzed by measuring the tension and displacement during loading.

When analyzing the mechanical properties of bolted joints, several factors need to be considered. The first is the material characteristics of bolts and connected parts; different materials have different yield strength, tensile strength, elastic modulus, etc.; these characteristics directly affect the performance of the joints under stress. Secondly, the geometry of the bolt, such as diameter, pitch and length, also has an important impact on its carrying capacity. Bolts with larger diameters are usually able to withstand higher tensile forces, but also increase the weight and cost of the joints. In addition, the size of the preload is also a key factor. Appropriate pre-tightening force can increase the frictional force of bolted joints and improve the stability and load-bearing capacity of the connection. However, too much preload may lead to premature yield of bolts or connected parts, and too little will not give full play to the performance of the joints. For the measurement and analysis of displacement, the deformation of the bolt joints during the stress process can be understood. By comparing the displacement changes in different loading stages, whether the joints are in the elastic stage or have entered the plastic stage can be determined.

**Figure 5:**
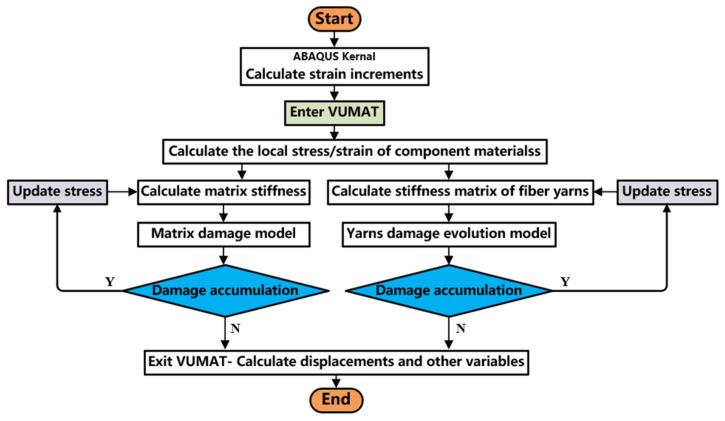
Calculation flowchart of the damage evolution model.

In the process of analyzing the failure of laminates, the constraints between bolts and nuts can be set, and it can be considered that there is no relative displacement between bolts and nuts, ignoring their damage, because in practical applications, bolts and nuts are usually designed to be strong enough to withstand the expected load without significant damage. The cohesive model is used to simulate the interface behavior between single-layer laminates. This model can accurately simulate the strength, stiffness and damage process of the interface by introducing a special cohesion unit at the interface. The cohesive model is suitable for studying the initiation, propagation and final failure of delaminated damage. The performance of the material plate is shown in Table 2. The VUMAT subroutine is compiled based on ABAQUS 6.14 software; calculation flowchart of the damage evolution model is shown in Figure 5.

## 5. Results and Discussion

In order to verify the reliability of the model and in accordance with the requirements of ASTM D7332-2009B, the experimental research on the bolt connection of the convex and counterhead of the pull composite material was carried out, the experimental data were recorded and the experimental results were analyzed.

### 5.1. Pullout Convex Head Model

**Figure 6:**
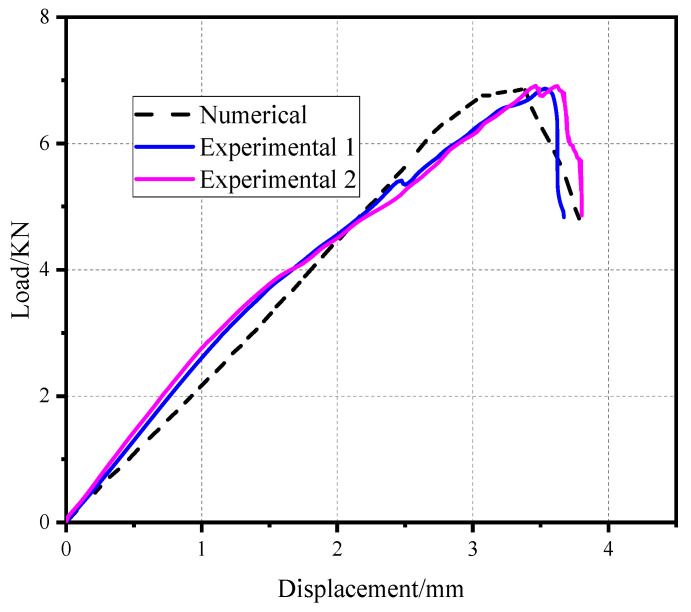
Load and displacement curve of the pull-out convex head model.

The material of the composite plate ply [−45/45/−45/45/45/−45] had a thickness of 2.28 mm and a single layer thickness of 0.19 mm. The load-displacement curve of the pull-out head model was compared with the numerical load-displacement curve, as shown in Figure 6. The experimental load-displacement curve is in good agreement with the numerical load-displacement curve, which shows that the numerical simulation model can accurately simulate the actual loading process of the mechanical behavior. The average values of the experimental results and numerical simulation results are 6.50 KN and 6.82 KN, respectively. The standard deviation was 4.92%.

**Figure 7:**
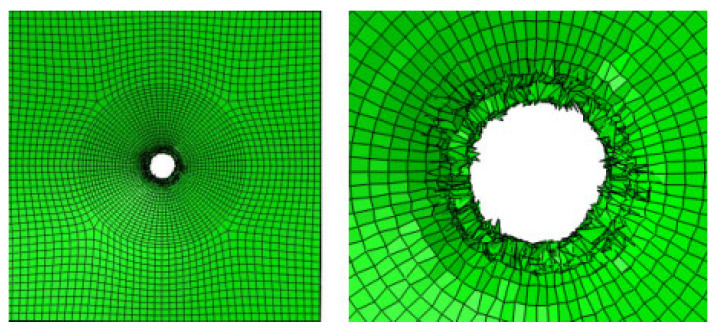
Deformation diagram of the pull-out convex head model.

**Figure 8:**
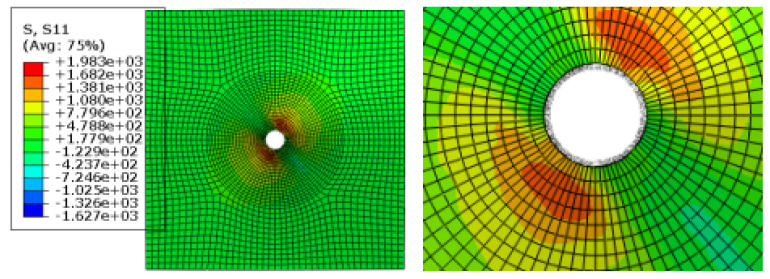
Stress nephogram of the pull-out convex head model.

As is shown in Figure 7, there is a gap between the composite plate and the fastener; the friction force between the fixture and the specimen is used to transfer the load in the initial stage. When the composite plate is in full contact with the bolt, the slope of the curve increases obviously. At this time, the plate starts to bear the load, and the curve has an obvious upward convex trend. At this time, the composite plate has not been damaged; with the further increase of the load, the slope of the curve begins to decrease when a critical value is reached, and when the failure criterion determines the initial damage of the material, the overall performance of the material begins to decline, and the internal structure begins to deteriorate. When the curve enters a fluctuating state and the load increases to the maximum, the joint structure emits a dense and large breaking sound, similar to the occurrence of shear failure, and finally, the bolt joint structure loses its bearing capacity completely and pull-out failure occurs in the composite. The pull-out failure of composite bolted joints can be considered as a brittle failure. Figure 8 shows the stress nephogram of the pull-out convex head model.

**Figure 9:**
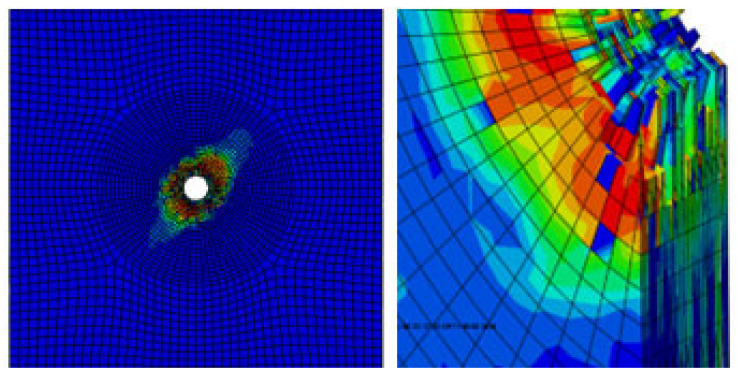
Damage output of the pull-out convex head model.

Figure 9 shows the damage of the components around the hole during the loading process of the convex head bolted joints. The stress distribution at the hole is relatively uniform, and the loading direction is perpendicular to the surface of the material plate. After the initial damage stage, with an increase in the load, these initial damages will begin to accumulate and gradually expand. During the process of increasing the load, the shear blocking phenomenon will occur inside the composite material plate. Because the fiber direction is perpendicular to the loading direction, the fibers may break due to overload, or the interface between the fibers and the matrix may debond. These damages will lead to a significant decrease in the bearing capacity of this layer.

**Figure 10:**
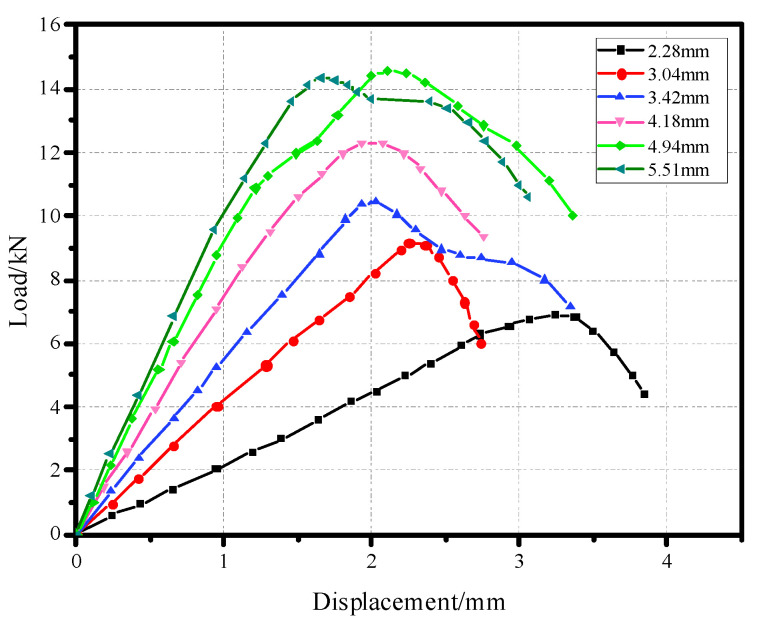
Displacement-load curves of different plate thicknesses of pull-out convex head models.

**Figure 11:**
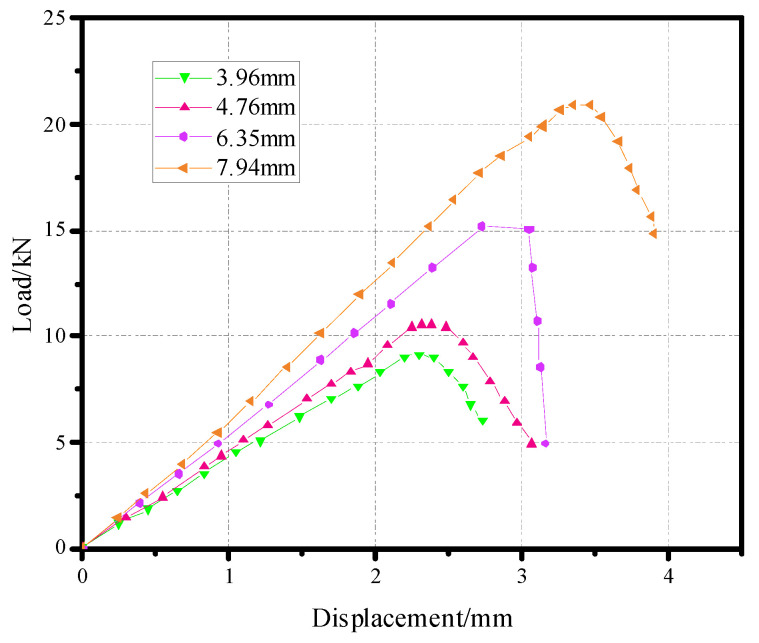
Displacement-load curves of different plate diameters of pull-out convex head models.

In order to explore the influence of the structural parameters of the material plate on the pull-out performance, different thicknesses and apertures of the material plate were set for the model, and the simulated displacement-load curve is shown in Figure 10 and Figure 11.

**Figure 12:**
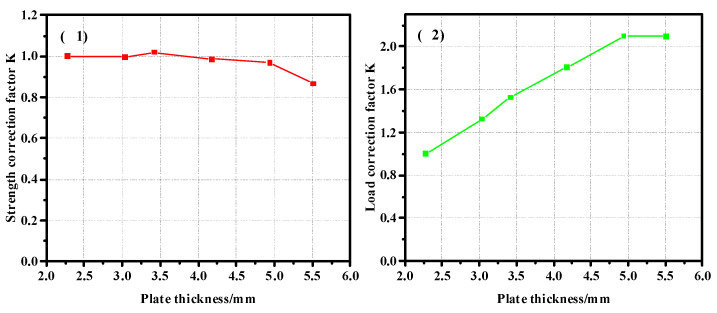
Thickness correction factor ((**1**). strength correction factor; (**2**). load correction factor) curve of the pull-out convex head model.

**Figure 13:**
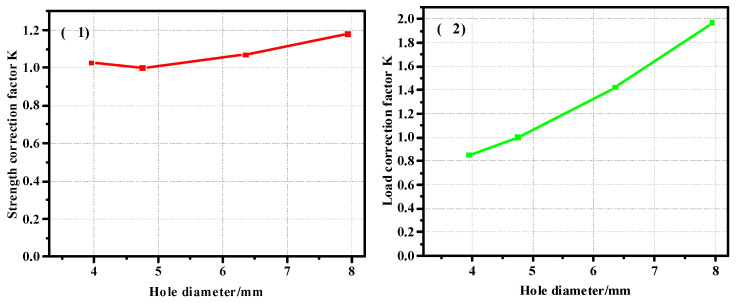
Diameter correction factor ((**1**). strength correction factor; (**2**). load correction factor) curve of the pull-out convex head model.

In order to obtain the correction coefficient of the influence of the thickness of the composite plate and the diameter of the nail hole on its strength, the model with a thickness of 2.28 mm was taken as the benchmark to calculate the correction coefficient, and the curve was drawn as shown in Figure 12. Figure 12(1) shows the correction coefficient curve of the strength value, and Figure 12(2) is the correction coefficient curve of the load value. It can be seen from the correction coefficient curve that when the thickness increases to a certain extent, its influence on the bearing capacity is almost zero. The model with a diameter of 4.76 mm was used as the benchmark to calculate the correction coefficient, and the curve was drawn as shown in Figure 13. Figure 13(1) shows the curve of the correction coefficient of strength value, and Figure 13(2) is the curve of the correction coefficient of load value.

### 5.2. Pullout Countersunk Model

**Figure 14:**
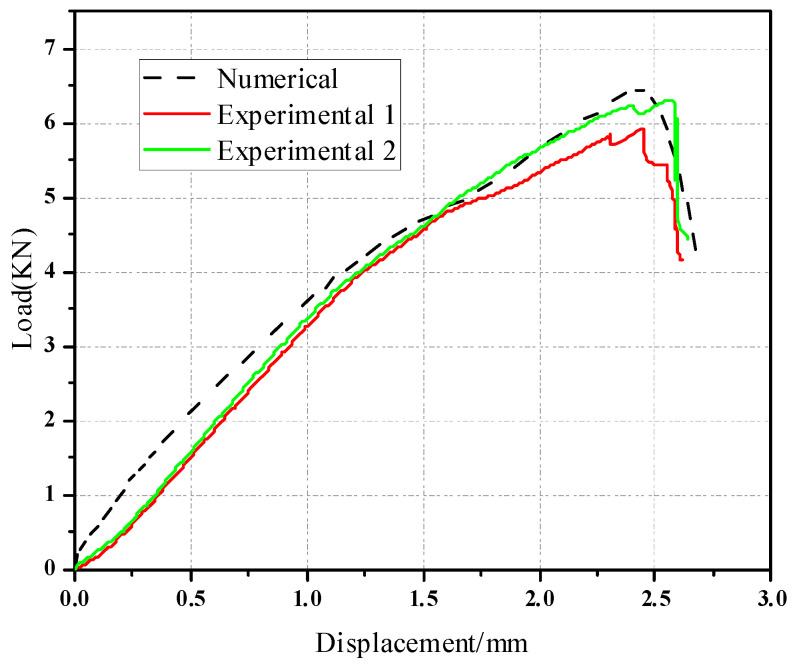
Pull load and displacement curve of the pull-out countersunk head model.

The material of the composite plate ply [−45/90/0/90/−45/45/0] had a thickness of 3.04 mm and a single layer thickness of 0.19 mm. Geometry refers to ASTM D7332-2009B. The experimental value and numerical comparison of the load-displacement curve of the specimen are shown in Figure 14. The calculated maximum load is 6.6 kN, the mean maximum load value is 6.01 kN and the relative error is 9.82%. It can be seen from the figure that the agreement is good.

Compared with the convex head bolted joints, the failure process of the countersunk bolted joints is very different. Owing to the special attributes of the countersunk bolted joints and the material plate, the composite laminate needs to cut the incision of the joints with the countersunk bolt on the surface, which induces pre-damage within the composite plate to a certain extent. Due to the unevenness of the incision, the unevenness of the joints between the bolt and the composite plate should be eliminated before loading the bolt. Therefore, the slope of the load-displacement curve in the initial test is greater than the simulated value, which is exactly opposite to the failure curve of the convex head bolted joints.

**Figure 15:**
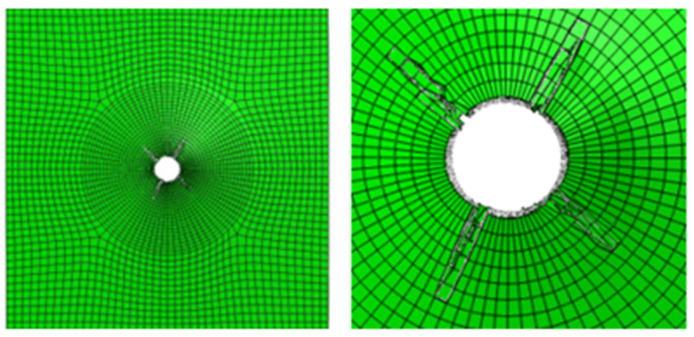
Deformation diagram of the pull-out countersunk head model.

**Figure 16:**
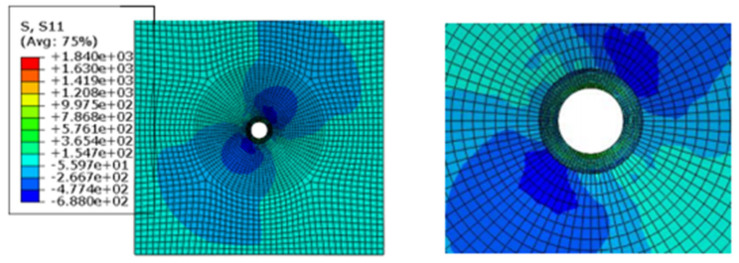
Stress cloud diagram of the pull-out countersunk head model.

It can be seen from the analysis of Figure 15 that in the countersink bolt foundation model, the stress concentration of the composite material may be mainly concentrated in the area where the bolt contacts the composite plate, which may cause a series of problems in the composites. First of all, it will make it easier for local materials to reach yield strength, which may lead to changes in the microstructure of the composite material, such as the interface between the fiber and the matrix and debonding, fiber fracture or matrix cracking. With the continuous action or increase of the load, these microscopic damages may gradually expand, further weakening the properties of the composite. The stress nephogram of the pull-out countersunk head model after loading is shown in Figure 16.

**Figure 17:**
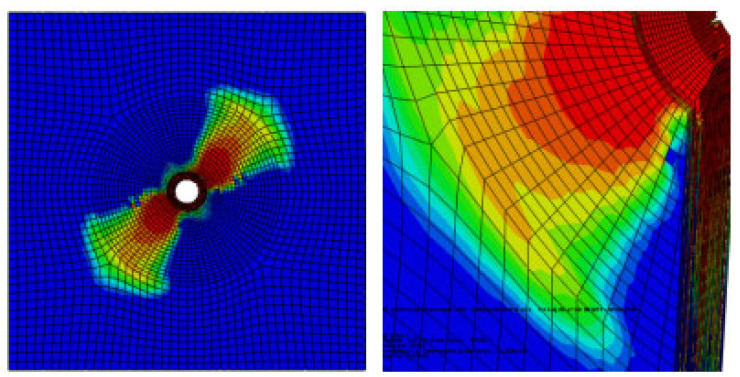
Damage output of the pull-out countersunk head model.

It can be seen from Figure 17 that the failure mode of the countersunk bolted joints is very different from that of the convex bolted joints. Compared with the convex bolted joints, the countersunk bolted joints will produce greater extrusion in the plane direction of the composite plate when bearing the load, and this extrusion action is concentrated in the area where the bolt and the composite plate contact. The composite material is subjected to strong compressive stress in this region. Due to the conical structure of the countersunk bolt, the stress distribution is not uniform. At the edge where the bolt head contacts the composite material, the stress concentration phenomenon is more obvious, which is also the first place where the damage easily occurs. Different from the shear failure of the convex head bolted joints, the countersunk bolted joints are subjected to extrusion failure, and the composite material near the bolt gradually fails from near to far, resulting in a significant increase in material plasticity, but this does not mean that the bearing capacity of the structure will always increase. Because the failure of the composite material will gradually weaken the overall performance of the connected structure, when the failure range expands to a certain extent, the connected structure will not be able to continue to bear the load, which will eventually lead to failure.

**Figure 18:**
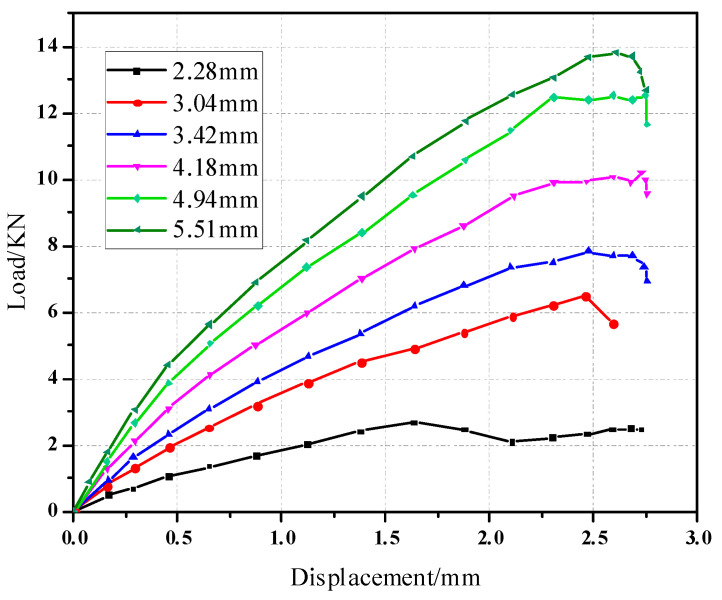
Displacement-load curves of different thicknesses of pull-out countersunk head models.

**Figure 19:**
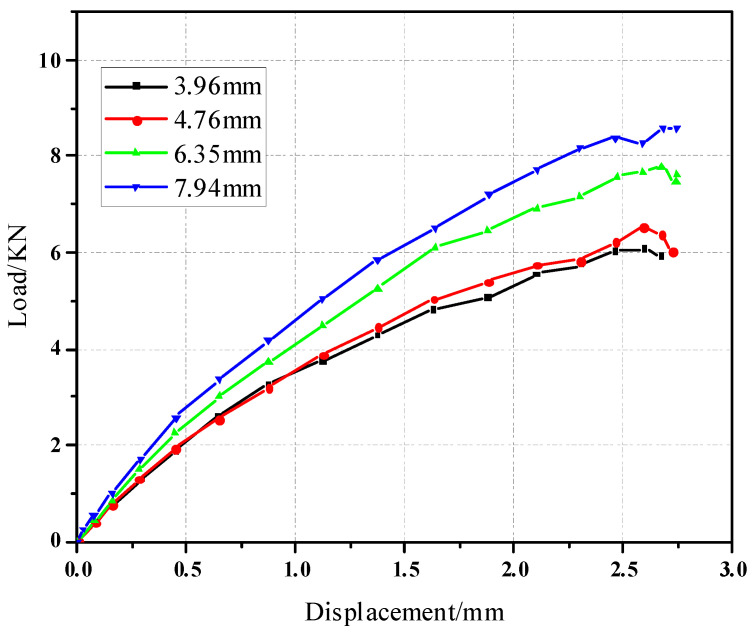
Displacement-load curves of different diameters of pull-out countersunk head models.

The pull-out curves of the countersunk bolt connection structure model with different thicknesses and apertures are shown in Figure 18 and Figure 19.

**Figure 20:**
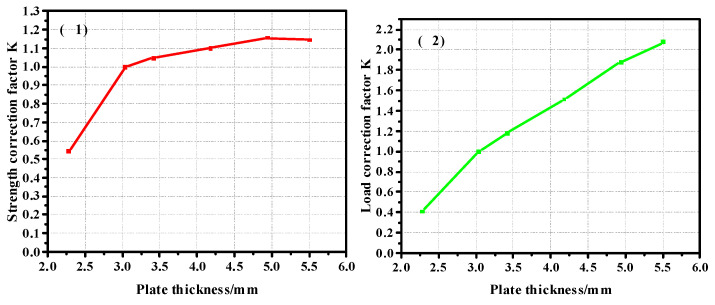
Thickness correction factor ((**1**). strength correction factor; (**2**). load correction factor) curve of pull-out countersunk head model.

**Figure 21:**
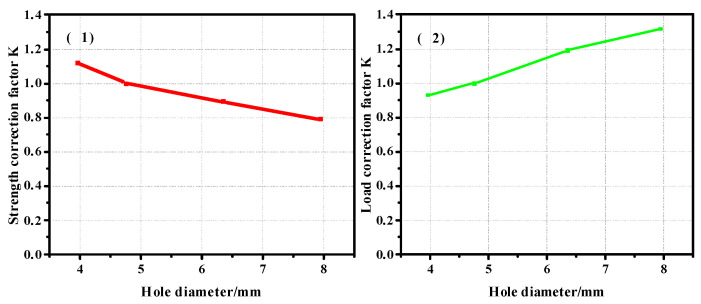
Diameter correction factor ((**1**). strength correction factor; (**2**). load correction factor)curve of pull-out countersunk head model.

In order to obtain the correction coefficient of the influence of the thickness of the composite plate and the diameter of the nail hole on its strength, the model with a thickness of 3.04 mm was taken as the benchmark to calculate the correction coefficient, and Curve 20 is drawn. Figure 20 is the thickness correction factor curve of the pull-out countersunk head model. Figure 20(1) shows the correction coefficient curve of the strength value, and Figure 20(2) is the correction coefficient curve of the load value. The diameter of 4.76 mm is taken as the basis to calculate the correction coefficient. Figure 21 shows the diameter correction factor curve of the pull-out countersunk head model. Figure 21(1) is the curve of the strength value correction coefficient, and Figure 21(2) is the curve of the load value correction coefficient.

## 6. Conclusions

In order to predict the failure behavior of composite laminate bolted joint structures, a detailed 3D finite element progressive damage modeling method is proposed. In addition, an experimental study is carried out on the joint structure of convex head bolts and countersunk bolts under tensile load, and the reliability of the proposed model is verified by comparing the experimental data with the predicted results. Some valuable conclusions can be drawn from this:

1. The model proposed in this paper can predict well the damage behavior of composite convex head bolt joints and countersunk bolted joints. The model proposed in this paper is used to analyze the composite lamination bolted structure with different plate thicknesses and apertures, and the correction coefficient of the influence of composite plate thickness and nail hole diameter on its strength is obtained.
2. Due to the difference of bolt forms, the failure forms of composite laminates are different in the joints structure under the action of out-of-plane load. Among them, the failure mode of the convex head bolt is closer to brittle failure, while the countersunk head bolt is closer to plastic failure. The convex head bolted joints have better ultimate strength than the countersunk bolted joints, and the countersunk bolted joints have better plastic properties. When the countersunk bolted joints are subjected to tensile load, the stress distribution is uneven due to the conical structure of the countersunk bolt. Stress concentration occurs at the edge where the bolt head contacts the composite material.
3. Through the work of this paper, the corresponding parameters of the bolted joint structure can be optimized in practical engineering so as to improve the overall safety and reliability of the structure, extend the service life of the structure and reduce the cost. The model proposed in this paper is not only applicable to the materials proposed in this paper, but also to other different material systems and layup structures.

## Figures and Tables

**Table 1:** The key parameters in the law of damage evolution.

*d_i_*_1_	*d_i_*_2_	*R_i_*_1_	*R_i_*_2_	*R_i_*_3_
0.75	0.90	20	25	30

**Table 2:** Material performance of material plate [33,34].

Material Property	Numerical Value
Longitudinal Young’s modulus, $E_{1}$ (GPa)	175
Transverse Young’s modulus, $E_{12}$ (Gpa), $E_{13}$ (Gpa)	8.05
Poisson’s ratio, $n_{12}$, $n_{13}$, $n_{23}$	0.32
Shear modulus, $G_{12}$ (Gpa), $G_{13}$ (Gpa), $G_{23}$ (Gpa)	4.37
Longitudinal tensile strength, $S_{1t}$ (Mpa)	2737
Longitudinal compressive strength, $S_{1c}$ (Mpa)	1602
Transverse tensile strength, $S_{2t}$ (Mpa) $S_{3t}$ (Mpa)	86.4
Transverse compressive strength, $S_{2c}$ (Mpa) $S_{3c}$ (Mpa)	212.8

## Data Availability

The original contributions presented in this study are included in the article. Further inquiries can be directed to the corresponding author.
